# Do sedentary behaviors mediate associations between socio-demographic characteristics and BMI in women living in socio-economically disadvantaged neighborhoods?

**DOI:** 10.1186/s12966-015-0209-1

**Published:** 2015-04-09

**Authors:** Sofie Compernolle, Katrien De Cocker, Gavin Abbott, Maïté Verloigne, Greet Cardon, Ilse De Bourdeaudhuij, Kylie Ball

**Affiliations:** Department of Movement and Sport Sciences, Faculty of Medicine and Health Sciences, Ghent University, Ghent, Belgium; Research Foundation Flanders (FWO), B-1000, Brussels, Belgium; Centre for Physical Activity and Nutrition Research, School of Exercise and Nutrition Sciences, Deakin University, Burwood, Victoria Australia

**Keywords:** Sedentary behavior, Adults, Socio-demographic factors, Mediation, Cohort study

## Abstract

**Background:**

Women living in deprived neighborhoods are a risk group for overweight and obesity, particularly during the childbearing years. Several socio-demographic characteristics may compound this risk, but little is known about why this might be the case. Sedentary behaviors are emerging as a socio-demographically patterned risk factor for obesity. The purpose of the present study was to assess socio-demographic differences in sedentary behaviors, and to examine whether these behaviors could explain the relation between socio-demographic variables and BMI (BMI) in this risk group.

**Methods:**

Women aged 18-46 years were recruited from 40 urban and 40 rural deprived neighborhoods in Victoria, Australia. In total, 3879 women reported socio-demographic variables (age, educational level, employment status, marital status, number of children, residential location and country of birth), sedentary behaviors (television time, computer time, total screen time and total sedentary time), physical activity, and height and weight, which were used to calculate BMI. For each socio-demographic variable, four single mediation models were conducted using two-level mixed-models regression analyses. Mediating effects were examined using the MacKinnon product-of-coefficients procedure and the Sobel test.

**Results:**

All socio-demographic variables were significantly associated with sedentary behaviors. Single mediation analyses revealed that television time (αβ = 0.017, 95% CI = 0.000, 0.030) and total screen time (αβ = 0.006, 95% CI = 0.000, 0.012) mediated 14.1% and 4.9% of the relationship between educational level and BMI, respectively. Total screen time mediated 45.1% of the relationship between employment status and BMI (αβ = -0.020, 95% CI = -0.033, -0.006), and television time mediated 8.2% of the relationship between country of birth and BMI (αβ = -0.008, 95% CI = -0.016, -0.001).

**Conclusion:**

Sedentary behaviors differed depending on socio-demographic characteristics, and partly explained the relationship between socio-demographic factors and BMI in this sample of women. Both television time and total screen time are potential behaviors to target in future programs aimed at reducing socio-demographic disparities in overweight and obesity.

## Background

During the last few decades, the prevalence of overweight (BMI (BMI) ≥ 25 kg/m^2^) and obesity (BMI ≥ 30 kg/m^2^) has reached epidemic proportions worldwide [1,2]. This poses drastic threats to public health, as overweight and obesity are related to several physical and mental health risks, such as cardiovascular diseases, type-2 diabetes, dementia, cancer and osteoporosis [3-6]. Therefore, action is urgently needed to reverse this negative trend and to reduce health risks related to overweight and obesity, in particular among at-risk population subgroups. An important at-risk group are women living in socioeconomically disadvantaged neighborhoods, particularly during the childbearing years, in which the likelihood of overweight and obesity is high [7-9].

Within this target group of women, overweight and obesity rates have been reported to be unevenly distributed depending on socio-demographic characteristics. For example, data from the Victorian READI study revealed that characteristics associated with higher BMI and greater risk of overweight included being older, married, less highly educated, and born in Australia [10,11]. Despite evidence of associations between socio-demographic factors and BMI, little information is available on the pathways through which socio-demographic factors may influence BMI in women. Previous studies have indicated that eating behaviors and physical activity explain only part of the link between socio-demographic factors and BMI [12-15]. Therefore, the role of other energy-balance related behaviors, such as sedentary behavior, could be important. Sedentary behavior can be defined as “any waking behavior characterized by energy expenditures ≤ 1.5 metabolic equivalents (METs), while in a sitting or reclining position” [16]. Prior research has demonstrated mixed results regarding the relationship between sedentary behavior and BMI [17,18]. Whereas some studies did not report a significant relationship between sedentary behaviors and BMI in adults [19,20], others have shown that increased sedentary time is related to a higher BMI, even among those who are sufficiently physically active [21-23]. In contrast to sedentary time, strong evidence was found for the relationship between television time and BMI [24-26]. Furthermore, sedentary behaviors tend to be socio-demographically patterned [27,28]. Those patterns are different for television time, computer time, and total sedentary time [27,28]; for example, younger women used computers more than older women, and older women spend more time watching television [27].

Consequently, the goal of this research was to gain insight into the mechanisms through which socio-demographic factors are associated with BMI in socio-economically disadvantaged women of childbearing age (18-46 years). The first aim of this study was to examine associations between socio-demographic factors and sedentary behaviors, as limited information is available on this relationship in women living in socio-economically disadvantaged neighborhoods. The second aim was to investigate whether sedentary behaviors mediated the potential associations between socio-demographic factors and BMI.

## Methods

### Procedure and participants

This study used baseline data of a longitudinal cohort study conducted between August 2007 and January 2008 in Victoria, Australia. The Resilience for Eating and Activity Despite Inequality (READI) study [29,30] was undertaken to investigate the resilience to obesity among socioeconomically disadvantaged women at childbearing age (18-46 years) and their children (5-12 years). The study protocol was approved by the Deakin University Human Research Ethics Committee, the Victorian Department of Education and the Catholic Education Office, and informed consent was obtained from each participant before the study started.

The sample for the READI study was randomly drawn from the electoral rolls of 40 rural and 40 urban Victorian neighborhoods, which were all classified as socioeconomically disadvantaged using the Australian Bureau of Statistics’ Socio-economic Index for Areas: Index of Relative Socio-Economic Disadvantage [30]. A total of 150 women per neighborhood were invited to participate. However, if an included neighborhood had less than 150 women within the predefined age range, all eligible women were invited. As a result, 11940 postal questionnaires were sent out, assessing the determinants of obesity-related behaviors, such as physical activity, sedentary behavior, and eating behavior, as well as the behaviors itself. Ten and twenty days after initially mailing, a first and a second reminder were sent. A total of 4934 (41%) women returned a completed questionnaire. Of these, 585 were excluded because (1) they moved out of the selected neighborhood (n = 571), (2) the questionnaire was not completed by the intended individual (n = 3), (3) they requested to withdraw from the study (n = 2) or (4) they were not in the predefined age group (n = 9). Data from women who were pregnant at the time of the survey (n = 210) were excluded from the analyses, as BMI was the outcome variable in the present study. Data from women who did not provide valid responses to questions on both height and weight were also excluded given that BMI is the outcome in all mediation analyses. After applying these exclusion criteria, the final sample included 3879 (32%) women.

### Measures

The READI questionnaire contained questions on body weight and height, socio-demographic characteristics, obesity-related behaviors (e.g. physical activity and sedentary behavior), and personal, social environmental and physical environmental determinants of these behaviors. The questionnaire items used in the present study are explained below.

#### Predictor measures: socio-demographic variables

Socio-demographic variables include age, educational level ([1] low-medium: ≤12 years, trade or certificate; [2] high: university or postgraduate), employment status ([1] unemployed: unemployed, keeping house and/or raising children full time, studying full time or retired; [2] employed: working full time or part-time), marital status ([1] alone: separated, divorced, widowed or never married; [2] married or in a relationship), number of children (none, one, two, three or more), residential location ([1] urban; [2] rural) and country of birth ([1] outside Australia; [2] Australia).

#### Potential mediators: sedentary behaviors

The following mediators were tested: television time, computer time, total screen time and total sedentary time. Total sedentary time was measured using the following questions: (1) ‘During the last 7 days, how much time did you usually spend sitting on a weekday (including the day and the evening)?’, and (2) ‘During the last 7 days, how much time did you usually spend sitting on a weekend day (including the day and the evening)?’. The questions are part of the long version of the International Physical Activity Questionnaire (IPAQ-L). This self-administered questionnaire showed excellent test-retest reliability (pooled r = 0.80) and acceptable validity (ρ = 0.34) in comparison with accelerometer-assessed sedentary time (CSA model 7164) in a 12–country study [31]. Based on the IPAQ scoring protocol, total sedentary time was calculated by summing the weekday minutes (multiplied by five) and the weekend day minutes (multiplied by two). The sum was divided by 420 to transform minutes/week into hours/day. Specific information on the activities undertaken while being sedentary was measured using some additional questions, which have been shown to be valid and reliable [32]. Participants were asked to report their time spent sedentary while watching TV, and while using a computer during the last seven days on weekdays as well as on weekend days. By summing the weekday minutes (multiplied by five) and the weekend day minutes (multiplied by two), and dividing the sum by 420 the following variables were calculated: daily hours spent sedentary while watching TV (television time), and daily hours spent sedentary while using a computer (computer time). These variables were summed to produce the amount of total screen time.

#### Outcome measure: BMI

Participants’ BMI was calculated by dividing self-reported weight in kilograms by the square of self-reported height in meters. Self-reported weight and height have shown good validity among Australian women [33].

#### Covariate: total physical activity

Because of its potentially strong relationship with socio-demographic characteristics, sedentary time and BMI, total physical activity was added as a covariate. Total physical activity was assessed using the long version of the reliable and validated IPAQ [31], in which both frequency (number of days in the last seven days) and duration (hours and minutes per day) of job-, transport-, domestic-, and leisure-time physical activity were assessed. By summing the physical activity per domain, total time spent physically active was calculated [34].

### Data reduction and statistical analyses

Raw data were used for descriptive statistics (see Table 1). The square root of the skewed variables (total television viewing time, total computer time, total screen time, total sedentary time, total physical activity and BMI) was calculated to improve normality and homogeneity of variances. Analyses were conducted using mixed models (SPSS Inc., Chicago, IL., USA) which included random intercepts for suburb-level variation. The following categories were used as reference categories in the analyses: having a high educational level, being employed, being married or in a relationship, living in a rural neighborhood and being born in Australia. All analyses were adjusted for total level of physical activity, and 95% confidence intervals (CI) were reported.

**Table 1:** **Socio-demographic characteristics, sedentary behaviors and BMI of the total sample**

**Characteristics**	**Total sample**
**Predictors: socio-demographic characteristics**	
Age (years), mean (SD) (n = 3830)	34.7 (8.2)
Employment status, n (%) (n = 3782)	
Unemployed	1188 (31.4)
Employed	2594 (68.6)
Educational level n (%) (n = 3831)	
Low-medium	2815 (73.5)
High	1016 (26.5)
Marital status n (%) (n = 3857)	
Married/ in a relationship	1349 (35.0)
Alone	2508 (65.0)
Number of children up to 18 years in household n (%) (n = 3809)	
None	1499 (39.4)
One	666 (17.5)
Two	993 (26.1)
Three or more	651 (17.1)
Residential location n (%) (n = 3879)	
Urban	1801 (46.4)
Rural	2078 (53.6)
Country of birth n (%) (n = 3866)	
Australia	3432 (88.8)
Other	434 (11.2)
**Potential mediators: sedentary behaviors (hours/day)**	
Television viewing time, mean (SD) (n = 3775)	3.6 (3.6)
Computer time, mean (SD) (n = 3615)	3.1 (4.2)
Total screen time, mean (SD) (n = 3585)	6.3 (4.7)
Total sedentary time, mean (SD) (n = 3749)	7.2 (4.8)
**Outcome**	
BMI (kg/m^2^), mean (SD) (n = 3879)	26.0 (6.1)

*SD* = Standard deviation.

**Figure 1:**
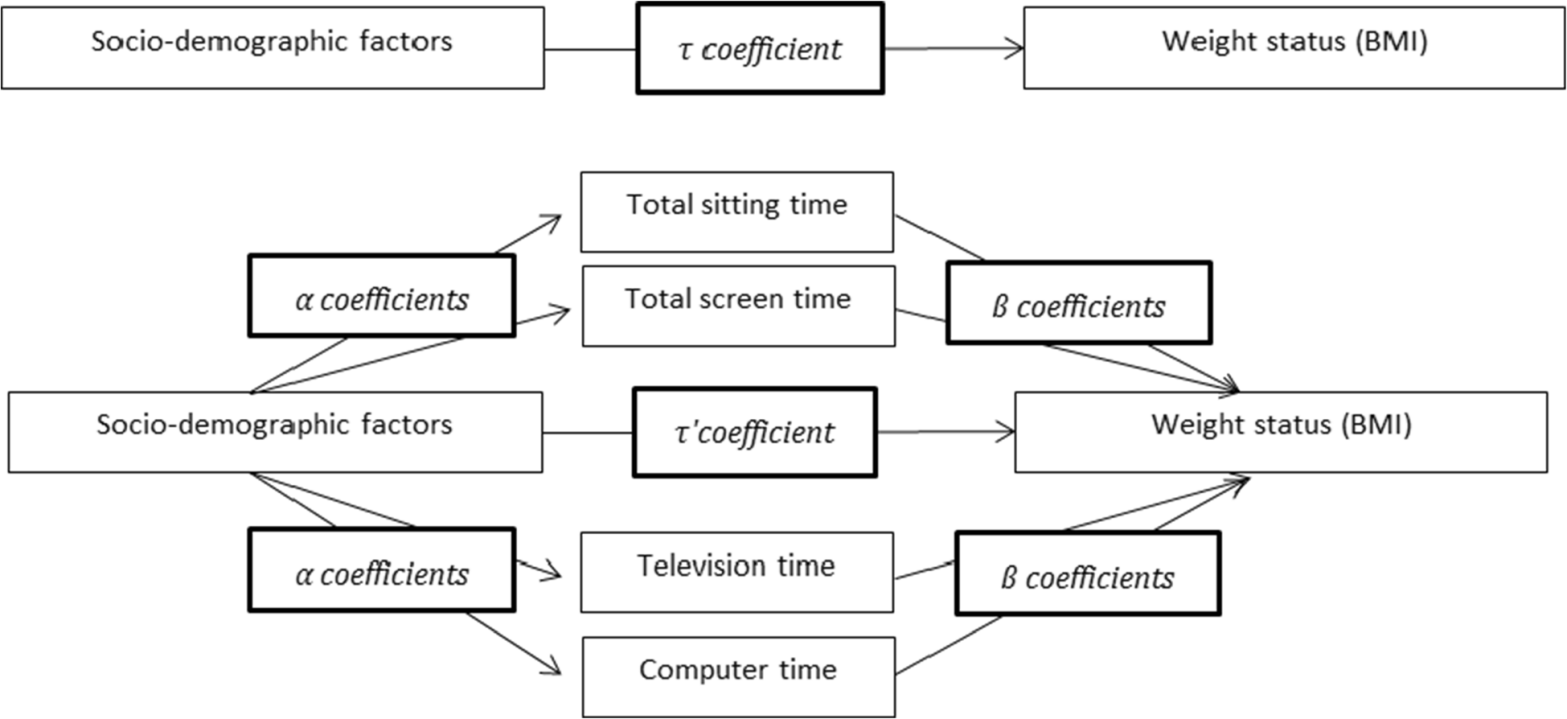
**Mediation model of the relation between socio-demographic factors and BMI as mediated through total sedentary time, total screen time, television time and computer time.** Represents the mediation model of the relation between socio-demographic factors and BMI as mediated through total sedentary time, total screen time, television time and computer time.

Firstly, the overall associations (τ-path) between socio-demographic factors and BMI were calculated by regressing the outcome variable (BMI) on each individual socio-demographic factor (age, educational level, employment status, marital status, number of children, residential location and country of birth) and the confounder (total physical activity) (see Figure 1). Subsequently, the mediating effects of television time, computer time, total screen time, and total sedentary time were examined using MacKinnon’s product-of-coefficients test in single mediation models [35]. The test includes the action theory test and the conceptual theory test. The action theory test estimates the association between each predictor and the potential mediators (α-path). The conceptual theory test estimates the association between each potential mediator and the outcome variable adjusted for the predictor variables (β-path). By multiplying the α-coefficient with the β-coefficient, the mediating effect (αβ) was calculated. This mediating effect (αβ) was only reported if the α- and β-path were significant, as MacKinnon states that a mediator needs to be significantly associated with both the predictor variable and the outcome variable [35]. Statistical significance of this effect was tested by dividing the product-of-coefficient (αβ) by its standard error (SE), which was calculated using the Sobel test [36]. The Sobel test was suitable as an alternative of bootstrapping because of the large sample size [36,37]. The proportion of the overall effect that was mediated was computed by dividing the product-of-coefficient (αβ) by the overall association (τ-coefficient).

## Results

### Participant characteristics

Characteristics of the total sample are presented in Table 1. The total sample consisted of 3879 women, with a mean age of 34.7 (SD: 8.2) years and an average BMI of 26.0 (SD: 6.1) kg/m^2^. The majority was employed at the time of the survey (68.6%) and about one-quarter had a high educational level (26.5%). Most women were not in a relationship (65.0%) and about sixty percent had at least one child. Additional participant characteristics are provided in Table 1.

### Main associations between socio-demographic factors and BMI (path τ)

All socio-demographic variables were significantly associated with BMI. Being older, having a low or medium level of education, being employed, being in a relationship, having more children, living in a rural neighborhood, and being born in Australia were associated with having a higher BMI (see Table 2).

**Table 2:** **Regression coefficients (τ) and 95% confidence interval (CI) of the main associations**

	**τ (SE)**	**95% CI**
Age	**0.013 (0.001)**	**0.011, 0.015**
Educational level (ref = having a high educational level)	**0.125 (0.022)**	**0.081, 0.169**
Employment status (ref = being employed)	**-0.044 (0.021)**	**-0.086, -0.002**
Marital status (ref = being married or in a relationship)	**-0.048 (0.021)**	**-0.088, -0.007**
Number of children	**0.024 (0.009)**	**0.006, 0.041**
Residential location (ref = living in a rural neighborhood)	**-0.098 (0.027)**	**-0.151, -0.045**
Country of birth (ref = being born in Australia)	**-0.197 (0.032)**	**-0.260, -0.134**

*SE* = standard error, *CI* = confidence interval, ref = reference category.

All significant associations are presented in bold font.

All analyses were adjusted for total physical activity.

### Associations between socio-demographic variables and potential mediators (path α)

As shown in Table 3 (action theory tests), all socio-demographic variables were significantly associated with computer time, total screen time and total sedentary time, except country of birth, which was not significantly related to computer time and total sedentary time. Only three socio-demographic variables were significantly associated with television time: educational level, number of children, and country of birth. Having a low or medium level of education, having less children and being born in Australia was associated with having a higher television time. Being younger, having a high level of education, being employed, not having a partner, having less children and living in an urban neighborhood was associated with having a higher computer time. Being younger, having a low or medium level of education, being employed, not having a partner, having less children, living in an urban neighborhood and being born in Australia was associated with having a higher screen time. Being younger, having a low or medium level of education, being employed, not having a partner, having less children and living in an urban neighborhood was associated with having a higher total sitting time.

**Table 3:** **Mediating role of sedentary behaviors on the association between socio-demographic factors and BMI**

**Single mediation models**	**Action theory tests** ^**a**^		**Conceptual theory tests** ^**b**^		**Mediating effects**		**Proportion mediated**
	**α (SE)**	**95% CI for α**	**β (SE)**	**95% CI for β**	**αβ (SE)**	**95% CI for αβ**	**%**
**Age**							
Television time	0.001 (0.001)	-0.002, 0.001	**0.188 (0.027)**	**0.134, 0.241**	-	-	-
Computer time	**-0.003 (0.001)**	**-0.006, -0.001**	**0.030 (0.021)**	**0.011, 0.070**	**-0.000 (0.000)**	-0.000,-0.000	**S**
Total screen time	**-0.002 (0.001)**	**-0.004, -0.001**	**0.177 (0.029)**	**0.120, 0.235**	**-0.000 (0.000)**	-0.001,-0.000	**S**
Total sedentary time	**-0.003 (0.001)**	**-0.005, -0.002**	**0.155 (0.034)**	**0.089, 0.221**	-0.000 (0.000)	-0.001, 0.000	-
**Educational level**							
Television time	**0.137 (0.014)**	**0.110, 0.165**	**-0.000 (0.000)**	**-0.000, -0.000**	**0.017 (0.006)**	0.000, 0.030	**14.1**
Computer time	**-0.015 (0.022)**	**-0.058, -0.028**	-0.000 (0.000)	-0.000, 0.000	-	-	-
Total screen time	**0.051 (0.014)**	**0.024, 0.078**	**0.151 (0.030)**	**0.093, 0.210**	**0.006 (0.003)**	0.000, 0.012	**4.9**
Total sedentary time	**0.025 (0.012)**	**0.003, 0.048**	**0.112 (0.034)**	**0.045, 0.179**	**0.004 (0.002)**	-0.001, 0.008	-
**Employment status**							
Television time	-0.011 (0.014)	-0.016, 0.038	**0.188 (0.028)**	**0.133, 0.242**	-	-	-
Computer time	**-0.270 (0.022)**	**-0.313, -0.288**	0.011 (0.022)	-0.032, 0.053	-	-	-
Total screen time	**-0.130 (0.013)**	**-0.155, -0.105**	**0.154 (0.031)**	**0.094, 0.214**	**-0.020 (0.007)**	-0.033, -0.006	**45.1**
Total sedentary time	**-0.074 (0.011)**	**-0.096, -0.053**	**0.120 (0.034)**	**0.052, 0.188**	-0.008 (0.004)	-0.016, 0.000	_
**Marital status**							
Television time	0.033 (0.013)	-0.007, 0.059	**0.184 (0.028)**	**0.130, 0.238**	-	-	-
Computer time	**0.110 (0.021)**	**0.070, 0.151**	0.024 (0.021)	-0.017, 0.065	-	-	-
Total screen time	**0.072 (0.012)**	**0.048, 0.097**	**0.167 (0.030)**	**0.109, 0.226**	**0.011 (0.004)**	0.003, 0.019	**S**
Total sedentary time	**0.077 (0.010)**	**0.057, 0.098**	**0.132 (0.034)**	**0.064, 0.199**	0.009 (0.005)	-0.000, 0.018	-
**Number of children**							
Television time	**-0.030 (0.006)**	**-0.041, -0.019**	**0.186 (0.028)**	**0.131, 0.241**	**-0.005 (0.002)**	-0.009, -0.002	**S**
Computer time	**-0.100 (0.009)**	**-0.117, -0.083**	**0.035 (0.022)**	**-0.007, 0.078**	**-**	-	-
Total screen time	**-0.063 (0.005)**	**-0.073, -0.053**	**0.182 (0.031)**	**0.122, 0.242**	**-0.010 (0.003)**	-0.017,-0.003	**S**
Total sedentary time	**-0.055 (0.004)**	**-0.064, -0.047**	0.144 (0.035)	0.076, 0.213	-0.006 (0.003)	-0.012,0.000	-
**Residential location**							
Television time	0.009 (0.014)	-0.019, 0.037	**0.185 (0.027)**	**0.131, 0.238**	**-**	-	-
Computer time	**0.082 (0.025)**	**0.034, 0.132**	0.023 (0.021)	-0.018, 0.064	-	-	-
Total screen time	**0.039 (0.014)**	**0.011, 0.066**	**0.165 (0.030)**	**0.107, 0.223**	**0.007 (0.003)**	0.001, 0.014	**S**
Total sedentary time	**0.037 (0.011)**	**0.015, 0.060**	**0.126 (0.034)**	**0.060, 0.193**	0.005 (0.003)	-0.001, 0.010	-
**Country of birth**							
Television time	**-0.062 (0.020)**	**-0.102, -0.022**	**0.173 (0.027)**	**0.119, 0.227**	**-0.008 (0.004)**	-0.016,-0.001	**8.2**
Computer time	0.015 (0.033)	-0.051, 0.080	0.019 (0.021)	-0.022, 0.060	-	-	-
Total screen time	**-0.052 (0.020)**	**-0.091, -0.014**	**0.150 (0.030)**	**0.091, 0.208**	-0.006 (0.004)	-0.014, 0.001	-
Total sedentary time	-0.034 (0.017)	-0.066, 0.001	**0.110 (0.034)**	**0.044, 0.176**	-	-	-

^a^Associations between socio-demographic predictors and mediators.

^b^Associations between mediators and BMI, adjusting for socio-demographic predictors.

*SE* = standard error, *CI* = confidence interval, *S* = suppression effect, – = mediation effects were not calculated if the α-path or the β-path were not significant, proportion mediated was not calculated if the mediation effect was not significant.

All significant associations are presented in bold font.

All analyses were adjusted for total physical activity.

### Associations between potential mediators and BMI (path β)

In all single mediation models, the conceptual theory tests revealed associations of television time and total screen time, with BMI (see Table 3). In six out of seven models, a significant relationship was found between total sedentary time and BMI, whereas only one model showed a significant relationship between computer time and BMI. All significant relationships were in the positive direction, such that greater amounts of sedentary activities were associated with higher BMI.

### Mediation effects

Estimated single model mediation effects are shown in Table 3. Both television time (αβ = 0.017; CI = 0.000, 0.030), and total screen time (αβ = 0.009; CI = 0.000, 0.012) mediated the relation between educational level and BMI, i.e. those who had a low or medium level of education reported more television time and more screen time, which was positively associated with a higher BMI. Total screen time (αβ = -0.020; CI = -0.033, -0.006) was a significant mediator of the relationship between employment status and BMI, i.e. those who were unemployed reported less television time, which was associated with lower BMI. Television time (αβ = -0.008; CI = -0.001, -0.016) mediated the relation between country of birth and BMI, i.e. those who were born overseas reported less television time, which was associated with lower BMI. The proportion mediated by television time and total screen time varied, with the highest proportion found for total screen time in the employment status model (45.1%), and the smallest proportion found for screen time in the educational level model (4.9%) (see last column of Table 3).

Significant suppression effects, which are expressed by opposite signs of the direct path (τ’-path) and indirect path (αβ-path), were found for four of the seven socio-demographic characteristics. Total screen time had a significant suppression effect on the relation of BMI with age, marital status, number of children, and residential location (e.g. older women reported less screen time, but had a higher BMI). Computer time had a significant suppression effect on the association between age and BMI, and television time and total screen time both had significant suppression effects on the relationship between number of children and BMI.

## Discussion

To our knowledge the present study is the first to explore associations between socio-demographic factors and sedentary behaviors in women living in socio-economically disadvantaged neighborhoods, and to examine whether these sedentary behaviors mediate the association between socio-demographic factors and BMI. To date, few studies have examined the relationship between socio-demographic characteristics and sedentary behaviors [27,28]. Moreover, none of these studies have focused on women living in deprived neighborhoods, despite this population having increased risk of being overweight, due to their lack of physical activity, and their unhealthy eating patterns [7-9].

Results of the action theory test revealed that less educated women and women who were born in Australia reported higher levels of television viewing, which is in line with previous research conducted in a more general population [38-41]. Higher levels of computer time were observed for more educated women, employed women, younger women, women with fewer children, women not in a current relationship, and women living in an urban neighborhood. Prior studies testing this relationship in a broader population found similar results concerning the association with age and educational level [42-44]; however, mixed or no associations have been found with respect to employment status and computer time [25,40,45,46]. No comparable studies were found assessing relationships between computer time and marital status, number of children or residential location in adults. The results of the action theory test also indicated that women who generally sit a lot during the day tended to be younger, less educated, employed, residing in an urban area, not be in a relationship, and to have fewer children. These findings add new evidence on the relationship between socio-demographic variables and total sedentary time, given that previous research demonstrated mixed effects [27]. The only socio-demographic variable consistently related to total sedentary time in past studies is the number of children [47-49]. Adults without children tended to spend more time being sedentary than adults with children [47-49], which is consistent with the present findings and may reflect time pressures precluding more leisure-time sedentary behaviors among women with children. Based on the results of the action theory test, it can be concluded that, within our risk group, some subgroups spend more time being sedentary, and are therefore, potentially more susceptible to develop overweight and obesity. These subgroups should be the main focus of future programs aimed at reducing sedentary behavior.

In mediation analyses some of the associations between socio-demographic factors and BMI in women living in socio-economically disadvantaged neighborhoods were explained by sedentary behaviors. After controlling for physical activity level, both television time and total screen time mediated the relation between educational level and BMI. This suggests that women with a lower level of education were more likely to spend television or screen time, which may result in a higher BMI. Nevertheless, reverse causality cannot be ruled out due to the cross-sectional nature of the study: it may be that lower educated women have higher BMI, which may lead to greater television or screen time. This result is in line with previous findings [19,39,50,51], and could be explained by (1) the fact that lower educated women are more likely to have a physically demanding job [52], leading them to prefer sedentary leisure-time activities [53], and (2) the fact that lower educated women less often consider the advantages of being physically active [54,55]. Furthermore, total screen time was also an important mediator in the relation between employment status and BMI, explaining nearly 50% of this association. This reflected the combination of the strongly significant relationship between employment status and computer time (more computer time among employed individuals), and the significant positive relationship between television time and BMI. The relationship between employment status and computer time has been found in previous research [56,57] and could reflect that women with jobs spend long parts of their days working in front of a computer screen [57], while unemployed women may be more likely to be at home chasing after their children. The relationship between television time and BMI could be explained by the fact that television time often replaces time spent moderately physically active [58], and television viewing has been associated with snacking behavior [59] and fast-food consumption [60]. Finally, television time was a significant mediator of the relationship between country of birth and BMI. Women who were born in Australia tended to watch more television, which was significantly related to having a higher BMI. There is evidence that among women born outside of Australia, a longer time since immigration to Australia predicts a body weight and lifestyle behaviors more similar to those of Australian-born women [61]. Consequently over time the differences in sedentary time and BMI observed for non-Australian born women may attenuate.

Besides the mediation effects, significant suppression effects were observed in several models. For example, a significant suppression effect was observed for total screen time on the relation between age and BMI, as age was positively associated with BMI, but negatively associated with total screen time. Consequently, an inconsistent mediation model was found. This inconsistency was also observed for (1) screen time on the relationship between marital status, number of children, and residential location, and BMI, for (2) computer time on the relationship between educational level and BMI, and for (3) television time on the relationship between number of children and BMI. The high number of suppression effects point out that, although sedentary behaviors could explain some pathways through which socio-demographic factors affect BMI, sedentary behaviors are likely to be just one of the factors contributing to BMI. The relationship between socio-demographic factors and BMI is a complex issue, which is, in all probability, mediated by different lifestyle behaviors. In order to gain insight into the complete underlying mechanism, future researchers should include various behaviors affecting BMI in multiple mediation models and should control for clustering of behaviors.

When interpreting the results of this study, some study limitations need to be considered. The most important limitation was the cross-sectional nature of the data, which limits causal interpretation of our findings. Secondly, the data were self-reported, and hence prone to social desirability and recall biases. Previous research assessing the validity of self-reported sedentary behavior questionnaires showed mixed results regarding total sedentary time. Whereas some studies reported an overestimation of total sedentary behavior [62,63], others reported an underestimation of total sedentary behavior [64-66]. Television time and total screen time were generally underestimated in comparison with device-based measures [67]. Thirdly, despite sending reminders, the response rate was relatively low, which might result in a selection bias [68], for example with potentially more highly motivated women participating in the survey. Fourthly, women that did not provide valid height and weight data were excluded from the analysis, which could have influenced the results. A comparison analysis showed that women that did provide valid height and weight data were older, had a higher level of education, and were more likely to be in a relationship than those who did not (data not shown). Finally, given that only women aged between 18 and 46 years old, living in socio-economically disadvantages neighborhoods were sampled, results might only be generalizable to this specific group of populations. On the other hand, some strengths should also be noted. More than 4,000 women from socio-economically disadvantaged neighborhoods participated in the study, which is a substantial sample given the difficulty of reaching this target group. As a result of the large sample size, mediation analysis were conducted with high statistical power. Moreover, the use of reliable and validated questionnaires was a strength of this study.

## Conclusion

The present study showed that sedentary behaviors are unequally distributed among population subgroups of women living in socio-economically disadvantaged neighborhoods. Moreover, these sedentary behaviors appear to play an important role in the association between socio-demographic variables and BMI. In particular, television time and total screen time, as these two behaviors mediated the relationship between educational level, employment status, country of birth and BMI. Women who had a low or medium level of education, women who were employed, and women who were born in Australia reported more television and/or screen time, which in turn was related to higher BMI. Consequently, future obesity prevention approaches should be developed (1) focusing on at-risk subgroup populations, and (2) aiming at reducing television time and total screen time, rather than sedentary time in general.

## Abbreviations

BMI: Body mass index
READI: Resilience for Eating and Activity Despite Inequality
IPAQ: International Physical Activity Questionnaire
